# Controlled Assembly of CdSe Nanoplatelet Thin Films and Nanowires

**DOI:** 10.1021/acs.langmuir.3c00933

**Published:** 2023-08-10

**Authors:** Emanuele Marino, Zhiqiao Jiang, Thomas E. Kodger, Christopher B. Murray, Peter Schall

**Affiliations:** † Van der Waals−Zeeman Institute, University of Amsterdam, Science Park 904, 1098XH Amsterdam, The Netherlands; ‡ Department of Chemistry, University of Pennsylvania, 231 S. 34th St., 19104 Philadelphia, (Pennsylvania), United States; § Dipartimento di Fisica e Chimica, Università degli Studi di Palermo, Via Archirafi 36, 90123 Palermo, Italy; ∥ Physical Chemistry and Soft Matter, Wageningen University and Research, Stippeneng 4, 6708WE Wageningen, The Netherlands; ⊥ Department of Materials Science and Engineering, University of Pennsylvania, 3231 Walnut Street, 19104 Philadelphia (Pennsylvania), United States

## Abstract

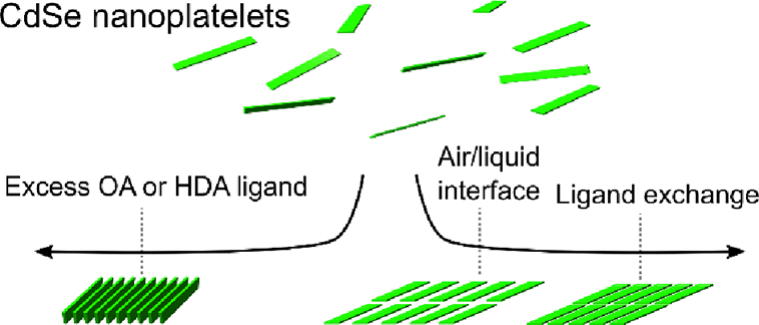

We assemble semiconductor CdSe nanoplatelets (NPs) at the air/liquid interface into 2D monolayers several micrometers wide, distinctly displaying nematic order. We show that this configuration is the most favorable energetically and that the edge-to-edge distance between neighboring NPs can be tuned by ligand exchange without disrupting film topology and nanoparticle orientation. We explore the rich assembly phase space by using depletion interactions to direct the formation of 1D nanowires from stacks of NPs. The improved control and understanding of the assembly of semiconductor NPs offers opportunities for the development of cheaper optoelectronic devices that rely on 1D or 2D charge delocalization throughout the assembled monolayers and nanowires.

## Introduction

Optoelectronic devices typically feature a 2D design. This type of architecture is ideal for 2D materials where charges travel freely in the plane of conduction. Traditional fabrication methods for these 2D materials employ slow and expensive techniques such as molecular beam epitaxy, atomic layer, or chemical vapor depositions, discouraging large-scale utilization.^1,2^ Recently, this scenario has dramatically shifted with the development of efficient and inexpensive routes for the synthesis of transition-metal dichalcogenide monolayers^3−5^ and semiconductor nanoplatelets (NPs).^6−11^

Semiconductor NPs are 2D colloidal nanocrystals characterized by a well-defined thickness of a few atomic monolayers, while their lateral dimensions are tunable up to hundreds of nanometers.^6^ Yet, to form the active layer of a macroscopic device, NPs must first be assembled into a thin film large enough to bridge the spacing between electrodes (micrometers to millimeters). While a variety of methods exists to drive the assembly of individual nanocrystals into films,^12,13^ the evaporation-driven assembly at the air/liquid interface represents a promising choice and has been used to produce ordered films of both isotropic and anisotropic nanocrystals.^14−18^ Yet, compared to 3D nanocrystals, the assembly of NPs requires special consideration since their larger surface-to-volume ratio introduces a strong bias for surface effects. Notably, the addition of antisolvent or oleic acid to a dispersion of NPs results in the formation of stacks of NPs.^19−22^ Instead, when assembling NPs at the air/liquid interface, surface tension plays an important role by discriminating between face-down or edge-up assembly.^22−24^ Overall, these observations call for a systematic study of the complex phase space of the superstructures generated from the assembly of NPs, ranging from edge-to-edge 2D thin films to face-to-face 1D stacks.^16,24−28^

While the assembly of NPs into superstructures is an active area of research, the pressing issue of their ligand exchange has also risen. NPs are nearly universally passivated by organic ligands consisting of aliphatic chains connected to a functional group bound to the surface, typically oleates (OA). These ligands are bulky and electrically insulating, requiring exchange with shorter ligands to achieve subnanometer interparticle distances that permit electrical conductivity in devices.^29−31^ Therefore, in addition to the reproducible assembly of NPs into 2D films, a ligand exchange method that does not disrupt film topology is needed.^31,32^

Here, we investigate the assembly of CdSe NPs at an air/liquid interface. We show that this procedure leads to the assembly of NPs into 2D thin films that extend over several micrometers. We assess the influence of ligand exchange on the topology of these NP films. We conclude that while the use of sulfonate groups disrupts the topology of the film, an exchange with short ligands stabilized by thiolate or carboxylate groups preserves long-range order across the film while decreasing inter-NP distance thus increasing density. Changes in the optical properties suggest that this optimized ligand exchange has the potential to improve the delocalization of charge carriers normal to the plane, generating an interest in the stacked assembly of NPs. We follow this interest to show that an excess of free ligands (>10^2^ per NP) induces the assembly of stacked NPs into 1D nanowires of several micrometers through depletion interactions.

## Materials and Methods

**Figure 1:**
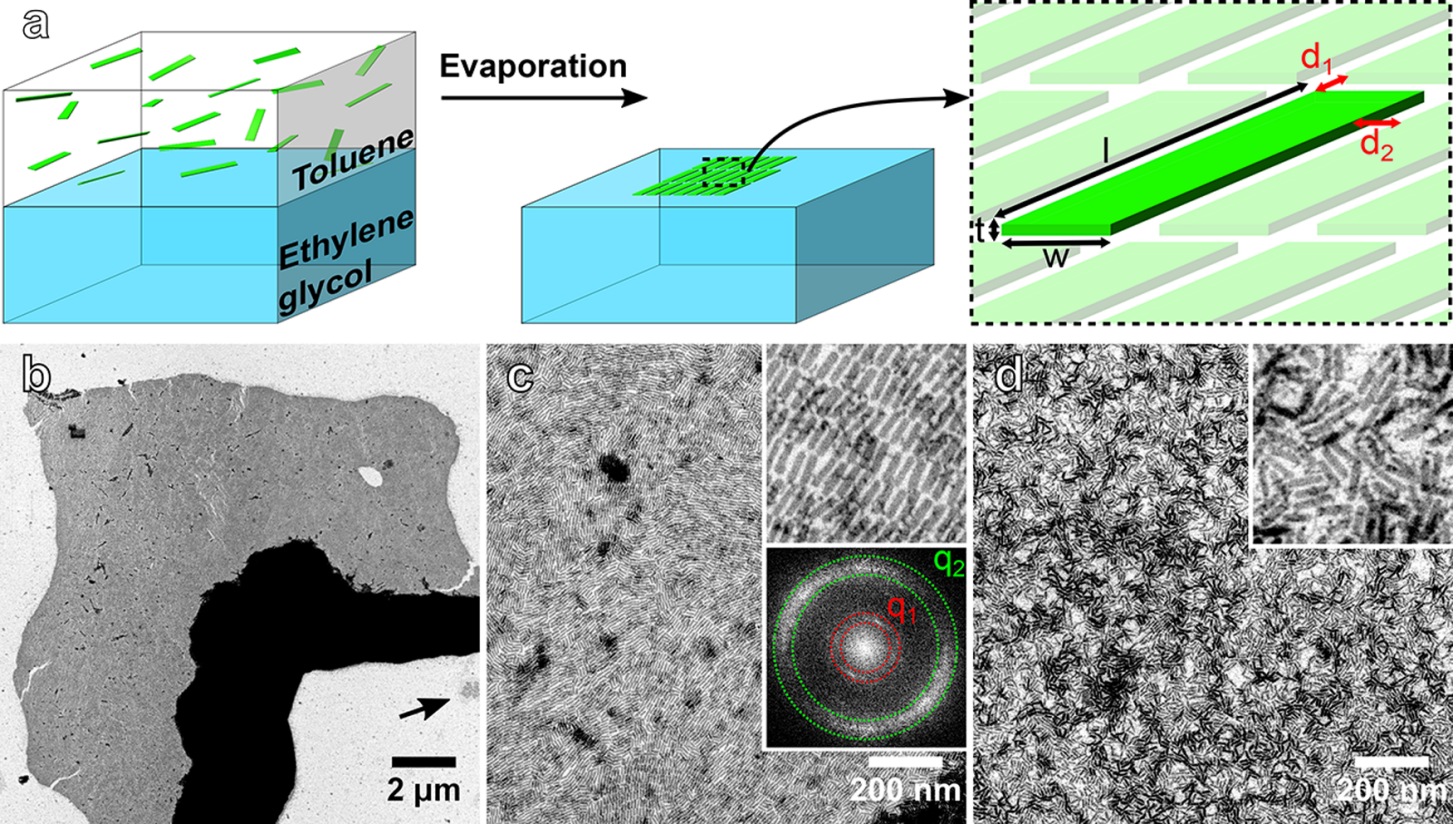
Nanoplatelet (NP) assembly at the liquid/air interface. (a) (Left) A dispersion of NPs in toluene is layered on ethylene glycol. (Center) Slow evaporation of toluene results in the face-down assembly of NPs at the air/liquid interface. (Right) Cartoon illustrating the symbols used in the manuscript, see also Table 1. (b) Low-magnification STEM micrograph of assembled NPs. Lighter areas correspond to a monolayer of NPs, while darker areas correspond to 3D aggregates. The arrow indicates a smaller patch consisting of NPs assembled in a disordered fashion. (c) Intermediate-magnification micrograph of NPs assembled in a monolayer. Top inset: 10-fold magnified detail of the monolayer. Bottom inset: Fourier transform of the original image indicating the main frequencies *q*_1_ and *q*_2_ discussed in the main text. (d) Intermediate-magnification micrograph of NPs assembled in a disordered fashion. Inset: 10-fold magnified detail of the superstructure.

We synthesize *l* = 21.3 ± 1.6 nm long, *w* = 5.6 ± 0.8 nm wide, and *t* = 1.2 nm thick CdSe NPs stabilized by OA ligands according to a procedure reported in the literature.^30,33^ The low aspect ratio of *l/w ≈* 3.8 was chosen to minimize NP twisting that can disrupt the assembly of 2D films.^20,30,34^Figure S1 shows a transmission electron micrograph of CdSe NPs of similar dimensions. The molar concentration of the NPs was calculated according to the procedure described in the Supporting Information.^35^ 1 mL of a 1.2 × 10^–8^ M NP dispersion in toluene was layered on 3.5 mL of ethylene glycol (EG) in a 40 mm glass Petri dish (Figure 1a). The Petri dish was immediately covered with a watch glass to slow down evaporation and left in the fume hood overnight. Subsequently, a ligand exchange was performed by adding a 200 mM solution of ligand in acetonitrile to the EG to obtain a final ligand concentration of 3 mM. The NP film was collected by placing a substrate in the subphase and gently raising it through the air/liquid interface. The choice of substrate ranged from carbon-coated TEM grids to glass substrates treated with 3-mercaptopropyl trimethoxysilane to improve wetting.^36^ The NP films were investigated using a Verios XHR SEM microscope (FEI) operated in transmission mode at 30 kV and 100 pA. The optical properties of the NP films were characterized using a Lambda 950 spectrophotometer (Perkin Elmer). The NPs were assembled into nanowires by adding an excess of OA to the dispersion in toluene, see below and the Supporting Information for additional details. This assembly process was followed in real time using a dynamic light scattering (DLS) setup (ALV) on the sample contained in an open 5 mm NMR tube. The laser beam (He:Ne, 633 nm) was aligned just above the meniscus of the EG/toluene interface. The sample was kept in the dark during the measurements to avoid photo-charging effects.^30,37^Table 1 introduces key parameters that recur throughout the text.

**Table 1:**
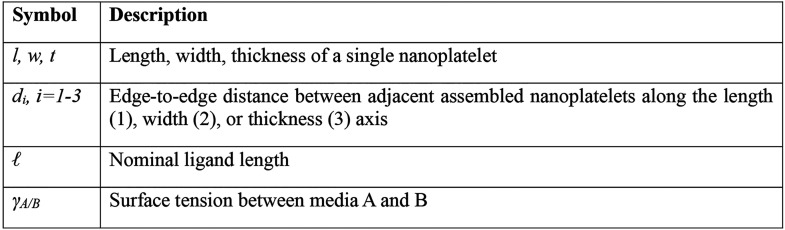
List of Recurring Parameters for This Manuscript

## Results and Discussion

### Nanoplatelet Assembly in 2D Thin Films

The microscopic investigation of the assembled film of NPs shows patches of several micrometers consisting of lower and higher contrast regions (Figure 1b). While the higher contrast regions consist of multilayers, a closer inspection reveals that the lighter regions consist of a monolayer of NPs lying face-down with the [001] crystallographic direction of CdSe normal to the substrate (Figure 1c). The assembled NPs show orientational and translational order, qualifying the structure as smectic with a grain size of a few hundred nanometers and an overall extension of 115 μm^2^.^38^ The Fourier transform of the image, shown in the bottom inset of Figure 1c, confirms the translational order, identifying two dominant frequencies. The lower frequency, *q*_1_ = 0.27 nm^–1^ corresponds to the center-to-center distance between adjacent NPs measured along their long-axes, resulting in an average edge-to-edge distance of *d*_1_ = 2π/*q*_1_ – *l* ≈ 2.0 nm (see schematic in Figure 1a). Meanwhile, the higher frequency, *q*_2_ = 0.77 nm^–1^ corresponds to the center-to-center distance between adjacent NPs measured along their short-axes, resulting in an average edge-to-edge distance of *d*_2_ = 2π/q_2_ – *w* ≈ 2.6 nm. An OA ligand can be approximated as a cylinder of length 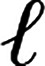 = 1.9 nm:^39^ Since 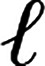 < *d*_1,2_ < 2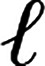, the OA ligands on adjacent NPs are either partially interdigitating or a low ligand surface coverage characterizes the side facets of NPs.^40^ Recent work supports the latter scenario since the weakest ligand binding sites are those in the immediate proximity of a facet edge.^41^ This observation suggests that ligands play a nontrivial role in the assembly of NPs.^39^ The NPs can also assemble in a disordered fashion, although less frequently (Figure 1d). This is typically the case for submicrometer patches such as the one marked by an arrow at the bottom-right edge of Figure 1b. If the rate of evaporation is too high, NPs may not have enough time to pack in an ordered fashion and aggregate as a kinetically trapped and disordered superstructure. Interestingly, most NPs still assemble with the [001] direction normal to the substrate as in the case of ordered superstructures.

Hence, irrespective of kinetics, the most favorable configuration of the NPs is the one maximizing the NP surface area in contact with EG. This result may seem unexpected: The hydrophobic OA ligands should minimize the contact with hydrophilic EG. Surface tension arguments can justify this apparent discrepancy. By lying flat, each NP replaces an area *A*_001_ = (*l* + *d*_1_)(*w* + *d*_2_) of air/EG interface with OA/EG interface, therefore saving *E*_001_ = (γ_air/EG_ – γ_OA/EG_)*A*_001_ ≈ 7 × 10^–18^ J ≈ 1800 *k*_B_*T* in interfacial energy, where γ_air/EG_ ≈ 47.7 × 10^–3^ J/m^2^ is the surface tension between air and EG and γ_OA/EG_ = [(γ_air/OA_)^1/2^ – (γ _air/EG_)^1/2^]^2^ ≈ 8.7 × 10^–3^ J/m^2^ is the surface tension between OA and EG,^42^*k*_B_ is Boltzmann’s constant, and *T* is the temperature. Instead, by laying on an edge facet of area *A*_–110_ = (*l* + *d*_1_)(*t* + *d*_3_), the same NP would save *E*_–110_ = (γ_air/EG_ – γ_OA/EG_)*A*_–110_ ≈ 5 × 10^–18^ J ≈ 1200 *k*_B_*T*, therefore yielding a higher energy configuration. These estimates are conservative since the area spanned by the ligands effectively decreases the ratio *A*_001_/*A*_–110_ from ∼4.7 to ∼1.5. Taking this into account, we propose the intervals of confidence *E*_001_ = 1100–1800 *k*_B_*T* and *E*_–110_ = 200–1200 *k*_B_*T*, which show that NPs laying face-down minimize the surface energy of the system.

#### Ligand Exchange and Film Topology

**Figure 2:**
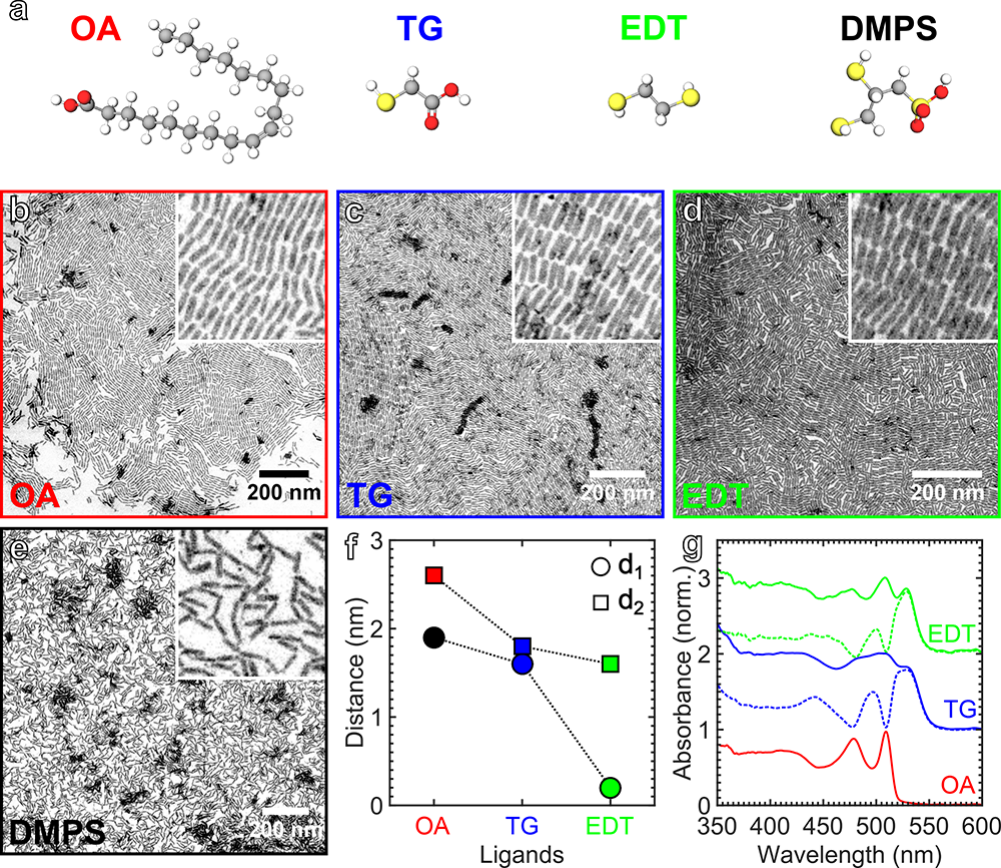
Effect of ligand exchange on ordered nanoplatelet (NP) superstructures. (a) 3D models of the ligands used. (b–e) Representative micrographs of assembled NPs stabilized by oleate (OA) ligands (b) and ligand exchanged with thioglycolate (TG) (c), 1,2-ethanedithiol (EDT) (d), and 2,3-dimercapto-1-propanesulfonate (DMPS) (e) ligands. Insets are magnified 10-fold. (f) Influence of ligand exchange on the edge-to-edge distances between neighboring NPs. Fourier transforms are shown in Figure S2. (g) Normalized light-absorption spectra of films of assembled NPs before and after ligand exchange (full lines) and their differences (dotted lines). Note: (f) and (g) do not report the data relative to ligand exchange with DMPS because this resulted in low-density, disordered films with light-absorption below the experimental detection limit.

To improve the coupling between NPs for applications involving electrical transport in optoelectronic devices, we replace the OA ligands with more compact molecules that allow for shorter (subnanometer) interparticle spacings. We test the influence of ligand exchange on NP films using 3 different, short thiolated ligands shown in Figure 2a: 1,2-ethanedithiol (EDT), thioglycolate (TG), and 2,3-dimercapto-1-propanesulfonate (DMPS). Ligand exchange with TG or EDT ligands preserves the smectic order of the NPs while resulting in denser films (Figure 2a–d). Instead, exchange with DMPS results in a less dense and disordered film (Figure 2e); we attribute this effect to the larger electron cloud of the sulfonate group providing a stronger electrostatic inter-NP repulsion. We extract the distances between neighboring NPs for the different ligands by analyzing the Fourier transforms of the images (Figure 2f and Figure S2). The exchange with TG and EDT decreases the inter-NP distance dramatically, reaching a shortest edge-to-edge distance along the short axis of *d*_1_ ≈ 0.2 nm after the ligand exchange with EDT.

To probe for possible coupling effects that can arise from ligand exchange,^43−45^ we measure the optical absorption spectrum of NP films, full lines in Figure 2g. The spectrum of NPs passivated with OA is characterized by a band-edge at 509 nm and sharp excitonic features. After ligand exchange, these features are substantially modified, the most apparent effect consisting of a red-shift of the band edge to 529 nm for both TG- and EDT-exchanged samples. We harness valuable information relative to this observation by taking the difference between the absorption spectra before and after ligand exchange, dashed lines in Figure 2g. The result consists in an absorption spectrum with features similar to the film before ligand exchange, but with all excitonic transitions red-shifted by 20 nm, or 92 meV. By taking the ratio between the absorbance values of the first exciton peak before and after ligand exchange, we estimate that 46% and 44% of NPs have been exchanged with TG and EDT, respectively. As discussed in the recent literature for other ligands,^32,46,47^ the exchange from longer to shorter ligands induces an expansion of the crystal structure of the NPs along the [001] direction. This expansion causes a decrease in the confinement energy of the charge carriers, leading to improved delocalization along the vertical axis of the NP, likely causing the observed red-shifts.^46,47^ Indeed, the observed magnitude of the red-shifts is consistent with other reports for aliphatic thiol ligands.^32,46−48^ This effect is likely convoluted with the delocalization of the electrons on the sulfur atom of the ligands, effectively increasing the thickness of the NP and relaxing quantum confinement.

### Nanoplatelet Assembly in 1D Stacked Superstructures

Besides the edge-to-edge assembly in a film, the NPs can also be steered to assemble face-to-face into fibers. In this configuration and under strong coupling conditions, charge carriers would travel freely from end to end of the stacks, acting as bottom-up assembled electrical leads. We attempt to induce the assembly of NPs into stacks through depletion interactions^20,21,49−52^ by adding an excess of 10^3^ OA ligands per NP to the dispersion in toluene prior to the layering on EG, resulting in the formation of OA micelles. When the distance between 2 NPs decreases below the size of a micelle, the interparticle volume becomes geometrically depleted of micelles. This causes an increase in the free volume available to the micelles and a decrease in the free energy of the system, resulting in a net attractive interaction known as depletion attraction.^42^

**Figure 3:**
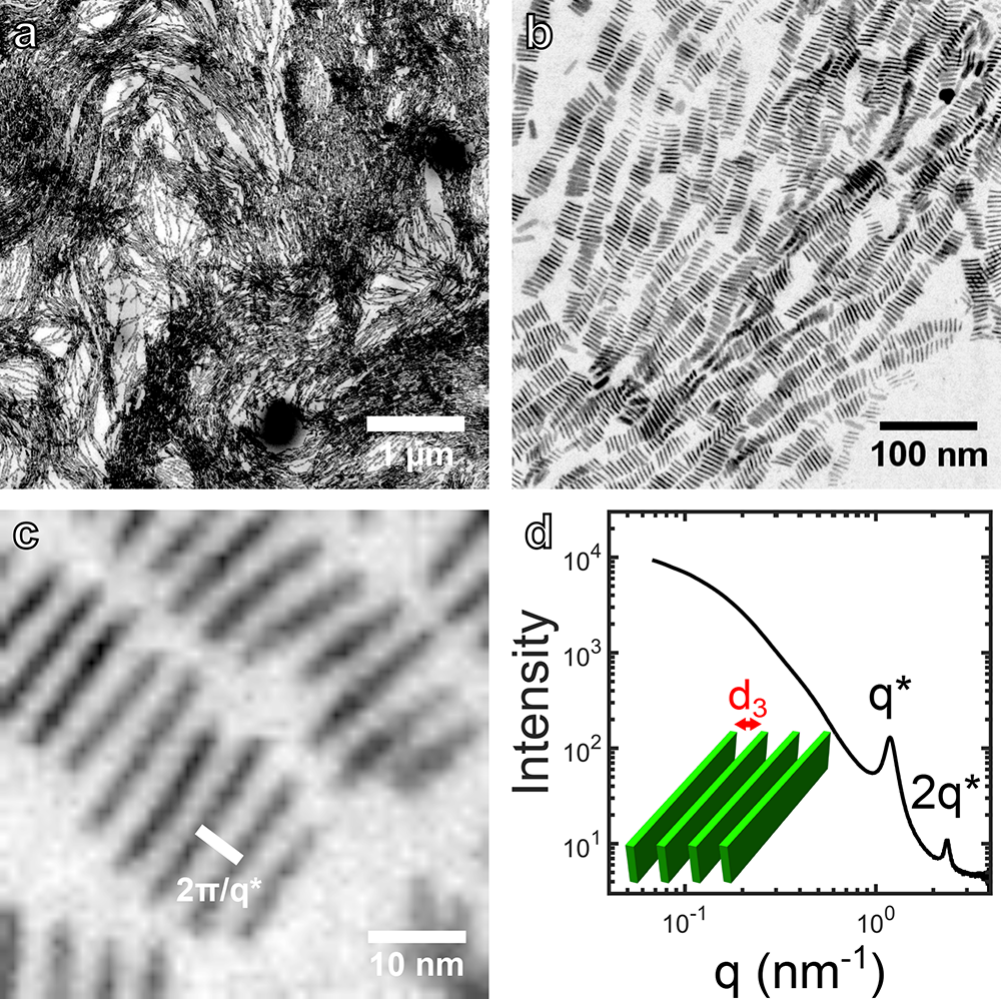
Nanoplatelet (NP) nanowires. (a) Low-, (b) intermediate-, and (c) high-magnification STEM micrographs of micrometer-long nanowires consisting of stacked NPs obtained by adding an excess of 10^3^ oleates (OAs) per NP in solution. (d) Azimuthally averaged small-angle X-ray scattering pattern of stacked NPs in toluene in the presence of excess OA ligands. The inset illustrates the nanoscale morphology of the NP stacks.

After complete evaporation of the hydrophobic phase, we observe that the NPs stack along the [001] direction to form nanowires, nanofibers,^53^ or nanofibrils,^25^ several micrometers in length (Figure 3a–c). These nanowires assemble into higher-order bundles that are reminiscent of supramolecular J-aggregates^54,55^ or mesostructures obtained from the oriented attachment of nanocrystals.^56,57^ The morphology of the nanowires is confirmed by small-angle X-ray scattering, revealing diffraction peaks at q* = 1.187 nm^–1^ and 2q*, Figure 3d. The positions of these diffraction peaks are indicative of a lamellar phase with a center-to-center distance of *t* + *d*_3_ = 2π/q* = 5.3 nm, that is consistent with the electron micrographs as shown in Figure 3c. Using *t* = 1.2 nm, we obtain the surface-to-surface distance *d*_3_ = 4.1 nm, which is slightly larger than twice the length of an OA ligand. Therefore, the surface-to-surface distance between neighboring NPs is larger between top facets than side facets, *d*_3_ > *d*_2_ > *d*_1_; this suggests that the ligand density is higher on the top facets than on the side facets.

**Figure 4:**
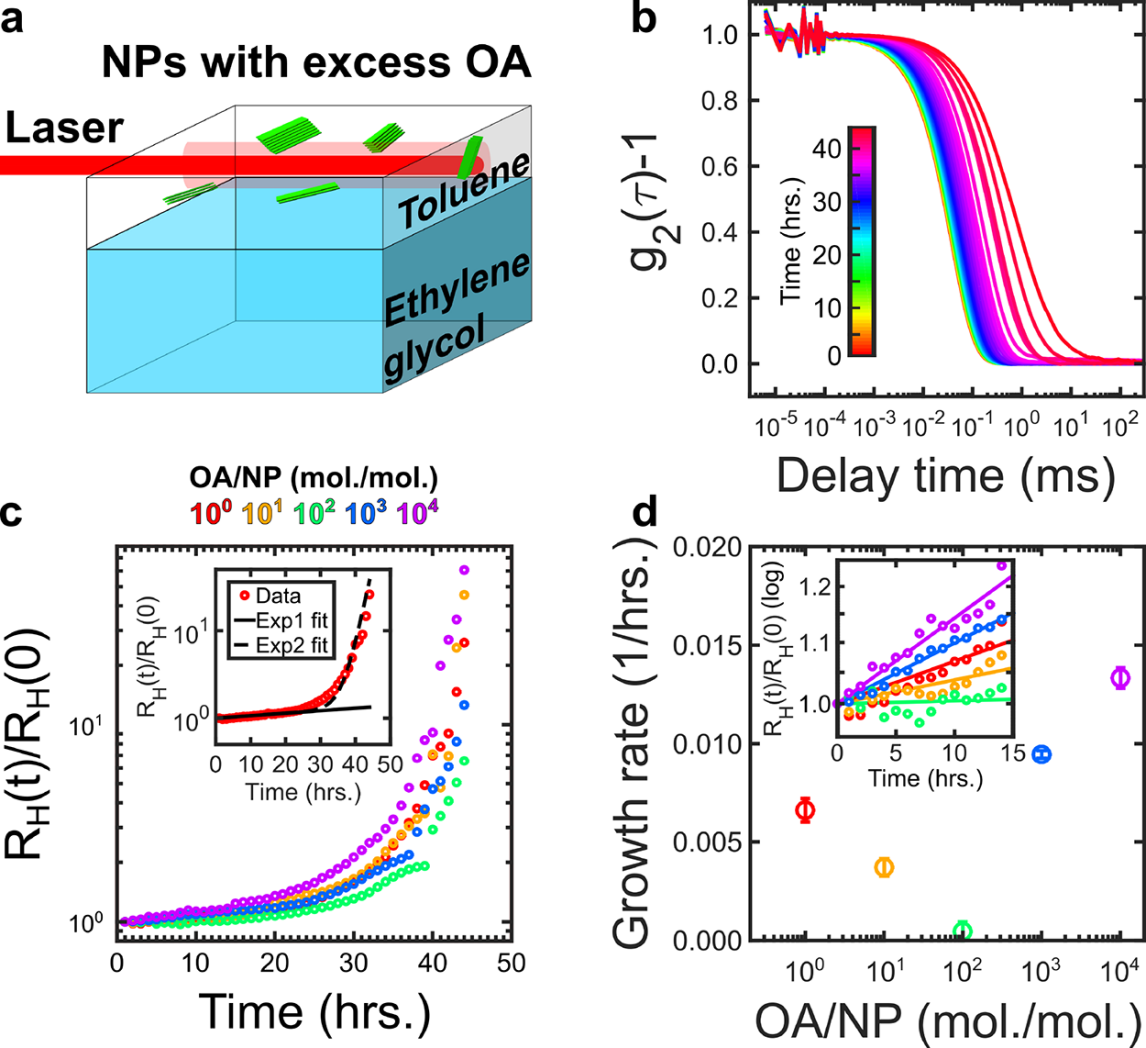
Nanoplatelet (NP) assembly kinetics in the presence of excess oleate (OA) ligands. (a) Schematic of the geometry of the measurement. (b) Scattered light intensity correlation function measured during the assembly for an excess of 10^2^ OA ligands per NP. (c) Normalized hydrodynamic radius as a function of assembly time for samples featuring 10^0^–10^4^ excess OA ligands per NP. Inset: Comparison between single exponential (Exp1 = exp(*G_i_t*)) and double exponential (Exp2 = exp(*G_i_t*) + exp[*G_f_*(*t* – *t*_0_)]) fits to the data for 10^0^ excess OA ligands per NP. Note the semilogarithmic scale. (d) Initial growth rate values (G_i_) as a function of excess OA ligands per NP. Inset: Initial increase of the normalized hydrodynamic radius as a function of assembly time for samples featuring 10^0^–10^4^ excess OA ligands per NP. The linear increase on a semilogarithmic plot suggests the presence of a single exponential. Single exponential fits (Exp1 = exp(*G_i_t*)) are shown as full lines.

To elucidate the formation of nanowires from individual NPs, we follow the assembly of NPs into nanowires in real time using dynamic light scattering (DLS). Usually, the kinetics of nanocrystal self-assembly are characterized using *in situ* small-angle X-ray scattering from brilliant synchrotron sources to achieve high temporal resolution and data quality.^39,58^ However, a table-top DLS setup should be able to follow the same assembly kinetics through changes in the hydrodynamic radius, *R_H_*. We study the assembly of NPs in proximity of the interface between EG and toluene as the latter evaporates by measuring the correlation function of the scattered light, *g*_2_(τ) (Figure 4a). The decay of the correlation function is due to the Brownian motion of the objects diffusing in solution. Specifically, the decay time of the correlation function, *t_c_*, is directly proportional to the hydrodynamic radius of the diffusing objects, *R_H_*, via *R*_H_ = (*k*_B_*T*/6πη)*q*^2^*t*_c_*,* where η is the solvent’s viscosity, *q* = (4πn/λ)sin(θ/2) the scattering vector, *n* is the solvent’s refractive index, θ = 90° is the scattering angle, and λ = 633 nm is the wavelength of the laser. During assembly, *t_c_* increases because of the increase in effective size of the NP assemblies, Figure 4b. By fitting each correlation function to an exponential decay with decay time *t_c_*(*t*), we can extract the change in *R_H_*(*t*) during assembly (Figure 4c). The initial hydrodynamic radius *R_H_*(0) = 22 nm is consistent with the size of NPs used, confirming that initially the NPs are well dispersed. We vary the number ratio of OAs per NP in a range from 10^0^ to 10^4^ to investigate the role of the excess ligand in the assembly process. For all samples, the hydrodynamic radius *R_H_*(*t*) increases as a function of time, reaching values 7–70 times larger than *R_H_*(0) (see Figure 4c). The initial increase can be fit with a single exponential function *R_H_*(*t*)*/R_H_*(0) *∼* exp(*G_i_t*), where *G_i_* is the initial growth rate (see the insets of Figure 4c, d). At later times, between 20 and 30 h, the values of *R_H_*(*t*) begin to increase faster than predicted by a single exponential. The addition of a delayed exponential leads to an improved fit, *R_H_*(*t*)*/R_H_*(0) ∼ exp(*G_i_t*) *+* exp[*G_f_* (*t* – *t*_0_)], where *G_f_* is the final growth rate and *t*_0_ is the delay time (see the inset of Figure 4c). These observations suggests that the self-assembly dynamics of NPs follow two main growth regimes.

Nanoparticle assembly can be characterized by diffusion- or reaction-limited kinetics.^59^ In the latter case, the average cluster size *R* increases exponentially in time: *R*(*t*) *= R*_0_ exp(*Gt*), consistent with our observations. This reaction-limited aggregation is described by a pair potential consisting of a repulsive barrier, which is the result of competing repulsive and attractive interparticle interactions; overcoming this barrier results in irreversible aggregation. We speculate that the barrier describing the assembly of NPs into nanowires results from the superposition of the steric repulsion between OA ligands bound to the surface of NPs with the combination of attractive van der Waals and depletion forces between NPs.

Indeed, the initial growth regime is sensitive to the amount of excess OAs per NP, as shown in Figure 4d: The growth rate *G_i_* first decreases, reaching a minimum at 10^2^ OAs per NP, and then increases. We interpret the initial decrease as the result of improved stabilization of the NPs following the addition of a small excess of ligand. However, the addition of a large excess of OAs can trigger the formation of micelles, which can cause attractive depletion interactions between the NPs. Indeed, this scenario is consistent with the observed increase in *G_i_* and the formation of small stacks of up to 1.2*l*/(*t* + *d*_3_) ≈ 5 NPs. While it is also conceivable that high ligand densities change the ligand monolayer and hence the ligand-mediated interactions, the sudden reversal from stabilizing to aggregating behavior suggests that another mechanism comes into play, which we associate with micelle-mediated depletion interaction.

The second growth regime follows with a delay *t*_0_ that does not depend on the amount of excess OA per NP, see Figure S3. Therefore, we propose that the second growth regime is controlled by the slow evaporation of toluene during the experiment. Indeed, this leads to an acceleration of the assembly dynamics at *t* > 30 h when most of the solvent has evaporated. The delay of ∼30 h of the second exponential, which is on the order of the evaporation time, shows the role of solvent evaporation in the acceleration of the assembly dynamics.

**Figure 5:**
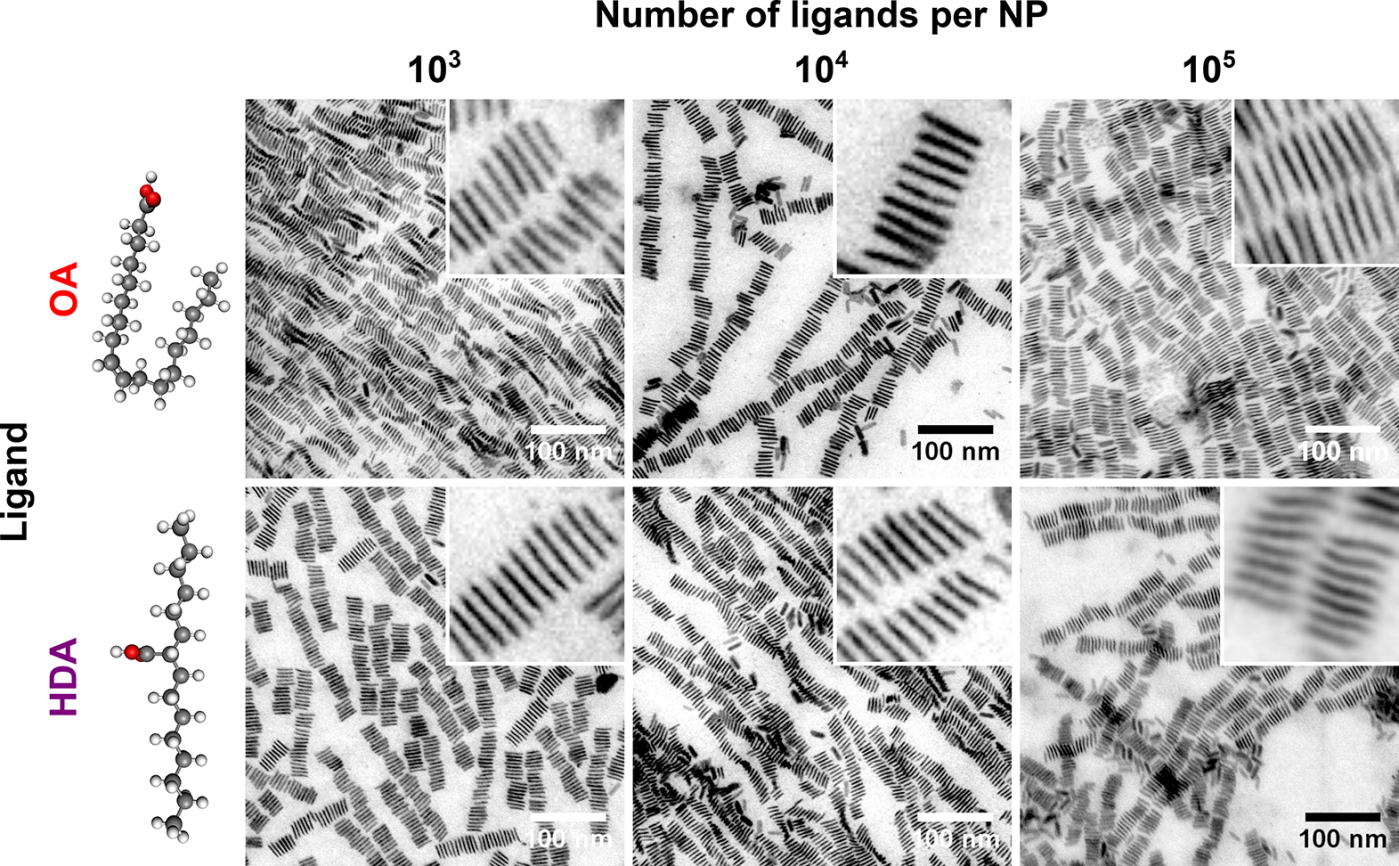
Influence of depletant branching. Nanoplatelets (NPs) assembled in the presence of the indicated excess of oleate (OA, top) and 2-hexyldecanoate (HDA, bottom) ligands. Both ligands result in similar stacked superstructures. Insets are ten-fold magnified.

The self-assembly into wires may also be affected by attractive ligand–ligand interactions resulting in ligand interdigitation.^39^ We investigate this effect by using a ligand with a branched structure, 2-hexyldecanoate (HDA). The branched morphology of HDA should minimize ligand interdigitation that could play a role in the assembly. Yet, microscopic investigation of NPs assembled in the presence of 10^3^–10^5^ excess HDA ligands per NP reveals the presence of nanowires very similar to those observed when using excess OA (Figure 5). Interestingly, the Fourier transform of the micrographs reveals that the center-to-center distance between adjacent NPs is larger for HDA than OA by about 4%, suggesting that partial ligand exchange from OA to HDA may take place concurrent to the assembly. We conclude that, in the presence of ligand excess, depletion attraction plays the leading role over ligand–ligand interactions in the assembly of NPs into nanowires.

## Conclusions

We have shown that the assembly of NPs at the air/liquid interface generates a rich phase-space of superstructure morphologies. NPs assemble edge-to-edge into patches several micrometers wide featuring smectic order with grains of hundreds of nanometers. We expect that further optimization may lead to grain sizes closer to the available surface area. Replacing the native OA ligand with shorter EDT or TG ligands results in a significant decrease in the edge-to-edge distance between neighboring NPs to reach sub-nanometer spacings without disrupting film morphology. This ligand exchange causes a delocalization of the wavefunction along the vertical ([001]) direction that we measure as a decrease in confinement energy of 92 meV. Since this larger delocalization may lead to novel applications in superstructures of stacked NPs, we exploit depletion interactions to drive the stacked assembly of NPs into nanowires several micrometers long. Using DLS to follow the assembly dynamics of NPs *in situ*, we observe reaction-limited growth dynamics, with growth rates governed by the ligand excess. The assembly takes place over 2 growth regimes that describe, respectively, the formation of small stacks and their assembly into longer wires. This assembly paradigm is robust with respect to the number of excess ligands per NP and the branched morphology of the ligand. We expect that improving our understanding of interparticle interactions and nanocrystal energetics may lead to electrically coupled nanowires consisting of confined-yet-connected NPs, 1D optoelectronic devices with an optical response based on the self-assembled state of colloidal nanocrystals. Significant opportunities may arise in the optical coupling of stacked NPs, as they are ideal candidates for sensing,^60,61^ lasing,^62−64^ superradiance,^65,66^ and superfluorescence^67,68^ emission.

## Supporting Information Available

Electron micrograph of CdSe nanoplatelets drop-casted on a carbon-coated TEM grid (Figure S1), electron micrographs and relative Fourier Transforms of edge-to-edge assembled nanoplatelets before and after undergoing ligand exchange (Figure S2), additional fitting parameters of the assembly kinetics (Figure S3), procedure to calculate the molar concentration of nanoplatelets and ligands, supporting references (PDF)
