# Supplementary material for: Controlled Assembly of CdSe Nanoplatelet Thin Films and Nanowires
Source: Langmuir. 2023 Aug 10;39(36):12533–40. doi: 10.1021/acs.langmuir.3c00933 (PMC10501200; doi:10.1021/acs.langmuir.3c00933)
Supplement: Supplementary file 1 — la3c00933_si_001.pdf [file la3c00933_si_001.pdf]

Supporting Information

Controlled Assembly of CdSe Nanoplatelet Thin-films and Nanowires

Emanuele Marino,[1,2,3,] Zhiqiao Jiang,[2,5] Thomas E. Kodger,[4] Christopher B. Murray,[2,5] Peter Schall[1,*]*

[1]Van der Waals–Zeeman Institute, University of Amsterdam, Science Park 904, 1098XH, Amsterdam, The Netherlands.

[2]Department of Chemistry, University of Pennsylvania, 231 S. 34th St., 19104, Philadelphia (PA), United States of America.

[3]Dipartimento di Fisica e Chimica, Università degli Studi di Palermo, Via Archirafi 36, 90123 Palermo, Italy.

[4]Physical Chemistry and Soft Matter, Wageningen University and Research, Stippeneng 4, 6708WE, Wageningen, The Netherlands.

[5]Department of Materials Science and Engineering, University of Pennsylvania, 3231 Walnut Street, 19104, Philadelphia (PA), United States of America.

*E-mail: emanuele.marino@unipa.it, p.schall@uva.nl

Supporting Figures

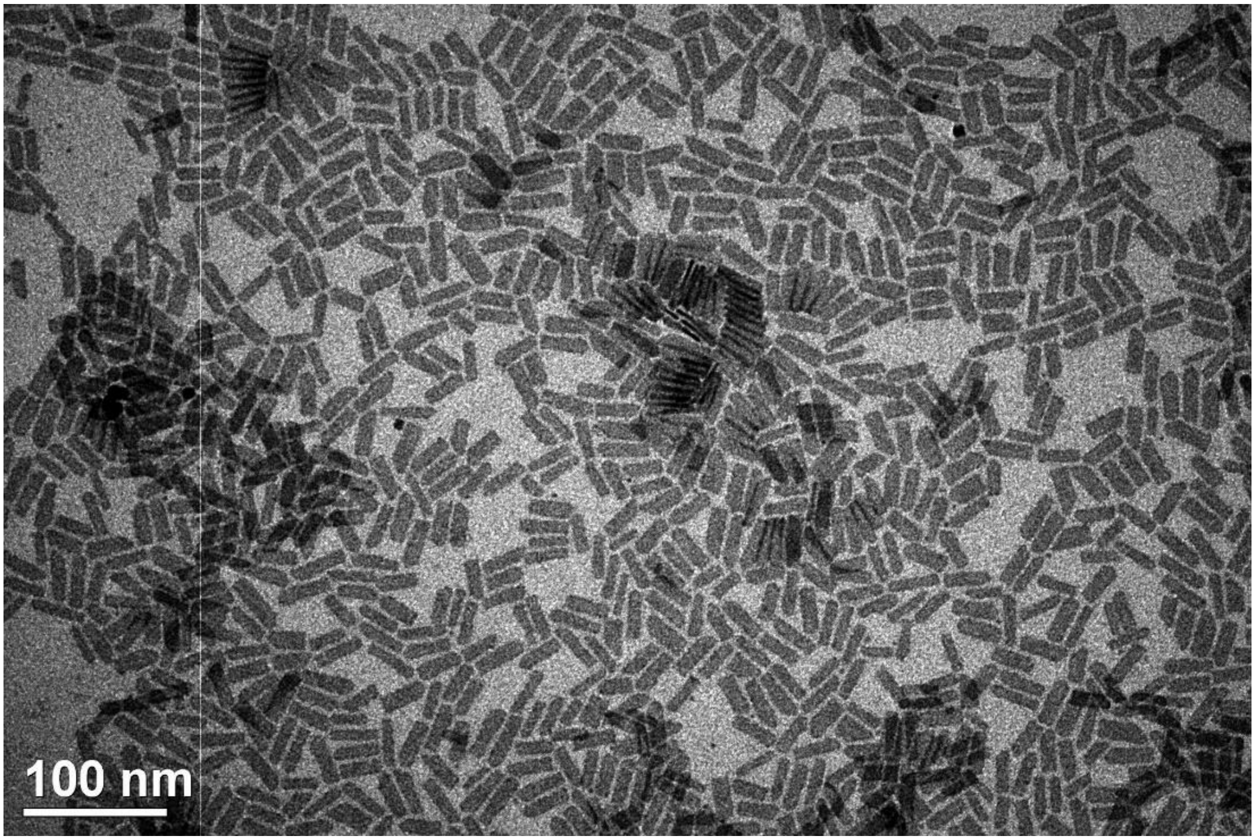

Figure S1: Transmission electron micrograph of CdSe nanoplatelets drop-casted on a carbon-coated TEM grid.

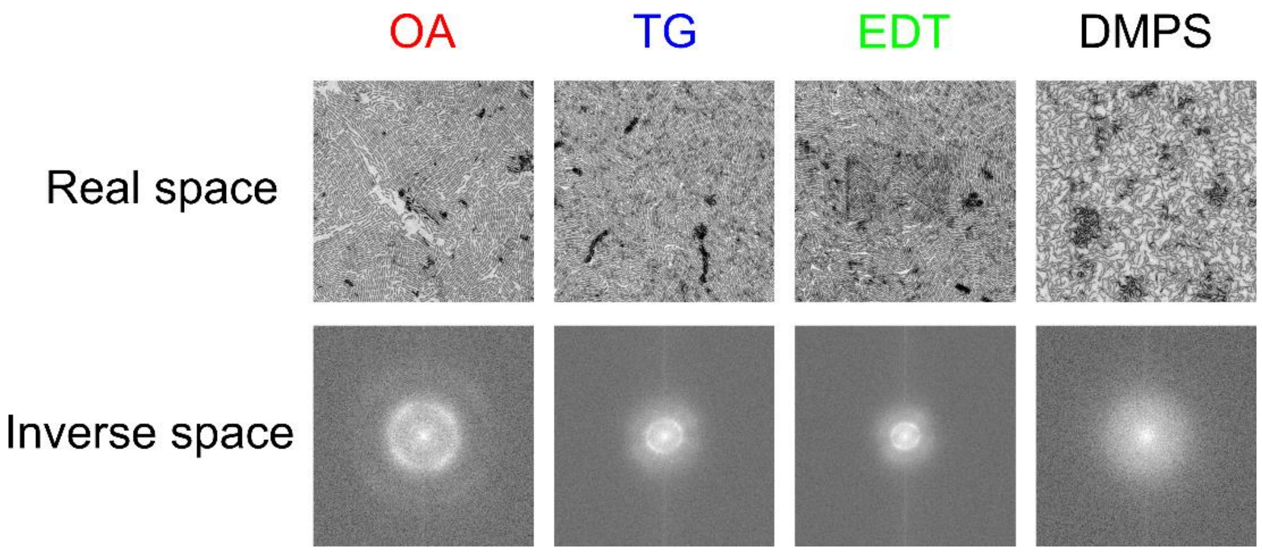

Figure S2: Real-space electron micrographs from Figure 2 (top row, 910x910 nm^2) and their Fourier Transforms (bottom row).

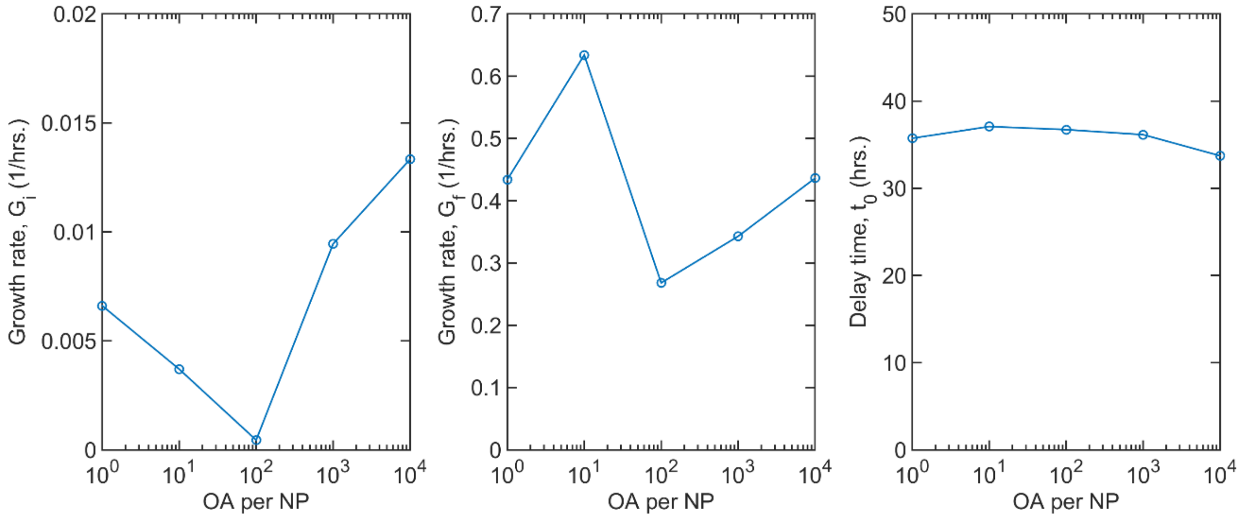

Figure S3: Fitting parameters from the data shown in Figure 4 of the manuscript using the fitting expression: $R_H(t)/R_H(0) = exp(G_i\, t) + exp[G_f\, (t-t_0)]$.

Supporting Text

How calculate the number of ligands per NPs:

First, we calculate the molar concentration of nanoplatelets with a known core volume:

$V_{CdSe\text{-}NP} = l*w*t$.

To do so, we start by measuring the absorption spectrum of a dispersion of nanoplatelets, $A_{CdSe\text{-}NP}(\lambda)$. According to the literature:[1]

"...the density of states is approximately constant at 350 nm and the absorbance is indicative of the number of unit cells present rather than the oscillator strength of a particular electronic transition."

Therefore,

$A_{CdSe\text{-}NP}$ (350 nm) $\propto N_{CdSe\text{-}NP}$,

where $N_{CdSe\text{-}NP}$ is the number of unit cells of CdSe NPs. We compare this value to that measured for a dispersion of spheroidal CdSe nanocrystals with a known core volume:

$V_{CdSe\text{-}NC} = (4/3)*\pi*r_{NC}^3$,

$A_{CdSe\text{-}NC}$ (350 nm) $\propto N_{CdSe\text{-}NC}$.

We then calculate the ratio:

$A_{CdSe\text{-}NP}$ (350 nm) / $A_{CdSe\text{-}NC}$ (350 nm) = $N_{CdSe\text{-}NP} / N_{CdSe\text{-}NC}$.

We use the sizing curves published in the literature (DOI: 10.1021/jp906827m or 10.1021/cm034081k) to calculate the molar concentration of the spheroidal CdSe NCs, $c_{CdSe\text{-}NC}$. We can now calculate the molarity of the NP dispersion if 1 NP had the same core volume as 1 NC ($V_{CdSe\text{-}NC} = V_{CdSe\text{-}NP}$):

$c_{CdSe\text{-}NP} = c_{CdSe\text{-}NC}*(N_{CdSe\text{-}NP} / N_{CdSe\text{-}NC})$.

However, in general NPs and NCs have different volumes ($V_{CdSe\text{-}NC} \neq V_{CdSe\text{-}NP}$), and therefore we need to correct for it. Therefore, the molar concentration of nanoplatelets within the dispersion amounts to:

$c_{CdSe\text{-}NP} = c_{CdSe\text{-}NC}*(N_{CdSe\text{-}NP}/N_{CdSe\text{-}NC})*(V_{CdSe\text{-}NC}/V_{CdSe\text{-}NP})$.

Once the molar concentration of nanoplatelets is known, we choose to add a certain number of ligands per NP (N_{OA}) by basic calculations. For instance, if the molar concentration of nanoplatelets is 12 nanomolar and we want to have 10^3 OA ligands per nanoplatelet (molecular weight 282.47 g/mol), then we need to reach a mass concentration of OA in toluene of:

$10^3*(12*10^{-9}$ mol/L)*(282.47 g/mol) ~ 3.4 mg of OA per L of OA/Toluene mixture.

We can convert this value to a volume ratio by using the density of OA, 0.895 g/mL:

$10^3*(12*10^{-9}$ mol/L)*(282.47 g/mol)/(0.895 g/mL) ~ 3.8 μL of OA per L of OA/Toluene mixture.

Supporting References:

*S*1. Leatherdale, C. A.; Woo, W. K.; Mikulec, F. V.; Bawendi, M. G., On the Absorption Cross Section of CdSe Nanocrystal Quantum Dots. *The Journal of Physical Chemistry B* **2002,** *106* (31), 7619-7622.
